# Relationship between anthropometric indices and Mizaj (temperament) in Persian medicine

**DOI:** 10.22088/cjim.14.2.205

**Published:** 2023

**Authors:** Mojgan Tansaz, Marjan Akhtari, Mohsen Naseri, Reza Majdzadeh, Mohsen Saberi Isfeedvajani, Mohammad Reza Shams-Ardakani, Fatemeh Nejatbakhsh, Abbas Ebadi, Morteza Mojahedi

**Affiliations:** 1 Department of Traditional Medicine, School of Traditional Medicine, Shahid Beheshti University of Medical Sciences, Tehran, Iran; 2 Department of Persian Medicine, School of Persian Medicine, Babol University of Medical Sciences, Babol, Iran; 3 Traditional Medicine Clinical Trial Research Center, Shahed University, Tehran, Iran; 4 Epidemiology and Biostatistics Department of Public Health School, Knowledge Utilization Research Center, Tehran University of Medical Science, Tehran, Iran; 5 Quran and Hadith Research Center and Department of Community Medicine, Faculty of Medicine, Baqiyatallah University of Medical Sciences, Tehran, Iran; 6 Department of Pharmacognosy, School of Pharmacy Tehran University of Medical Sciences, Tehran, Iran; 7 Behavioral Sciences Research Center, Nursing Faculty, Baqiyatallah University of Medical Sciences, Tehran, Iran; 8 Traditional Medicine and History of Medical Sciences Research Center, Health Research Institute, Babol University of Medical Sciences, Babol, Iran

**Keywords:** Anthropometric indices, Mizaj, Persian medicine, Personalized medicine, Temperament.

## Abstract

**Background:**

The process of diagnosis and treatment in Persian medicine (PM) are based on the concept of Mizaj (temperament). Among the indices of Mizaj determination, anthropometric indices are less influenceable regarding age change and other environmental factors. The purpose of this study was to investigate the relationship between anthropometric indices and Mizaj.

**Methods:**

Four PM experts determined the Mizaj of 121 participants. Those who had ≥70% agreement in their Mizaj determination by the experts were selected and their anthropometric indices were measured. The best cutoff point of each index and its relationship with the defined Mizaj were extracted using Receiver Operative Characteristic Curve and Binary Logistic Regression.

**Results:**

52 out of 121 participants entered the main study. The warm-Mizaj people had larger dimensions in height, shoulder, chest, palm and sole width, and head height. Cold-Mizaj people had smaller dimensions in weight, height, shoulder, chest and head. High levels of BMI, chest depth and head dimensions had the highest correlation with the wet Mizaj and lower dimensions of these indices had the highest correlation with the dry Mizaj.

**Conclusion:**

Among the anthropometric indices, chest, palm, sole dimensions, head height and weight had the highest correlation with warmness/coldness and BMI, head width and chest dimensions had the highest correlation with wetness/dryness. The BMI which is more closely related to the soft tissue, only correlates with the wetness/dryness, while, bone dimensions are associated with warmness/coldness. Further studies are needed to metricize the anthropometric indices for Mizaj determination.

Personalized medicine is a new aspect in modern medicine which considers the diagnoses and treatment of illnesses based on individual differences (1). Although it is a new topic in modern medicine, but it is the basis of disease diagnosis and treatment and prophylactic recommendations in most traditional medicines such as Traditional Chinese Medicine, Ayurveda and Persian (Unani) Medicine (2-7). In Persian medicine (PM) individual differences have been described as Mizaj (temperament) that is defined as a quality which is the final result of the reaction among the qualities of four elements (air, fire, water and soil) (8) which are warmness/wetness, warmness/dryness, coldness/wetness and coldness/dryness respectively and finally nine groups of Mizajes are described including four simple Mizajes (warm, cold, wet, dry), four compound Mizajes (warm/dry, warm/wet, cold/dry, cold/wet) and a temperate Mizaj (2). Every person is born with a basic Mizaj called "innate Mizaj". Under the influence of age change and lifestyle varieties, the secondary Mizaj is formed that may differ from the initial one.

Therefore, PM scholars have put these indices in "ten Mizaj determination criteria" including touch, muscle and fat mass, hair condition, skin color, physique, impressibility speed, sleep and wakefulness, physical functions, quality of waste matter (stool, urine, sweat) and psychological reactions (3, 9-14). Nowadays, Mizaj determination is commonly done by clinical findings without any standard metric tools, which are not quite reliable (8, 15). Considering that Mizaj determination is necessary for providing lifestyle recommendations and disease diagnosis and treatment (3, 9-12), finding standard tools for Mizaj determination seems essential (3). Since most of the initial Mizaj determination indices are influenced by environmental conditions such as nutrition, education, etc., focusing on indices that are less affected by these factors seems necessary, such as "physique" (anthropometric indices) and "muscle and fat mass" (obesity and slimming) which are more readily measurable and less affected by environmental factors and age changes than the other ones (6). According to PM, warm Mizaj results in large size skeleton, cold Mizaj in small size skeleton and moderate Mizaj in moderate size skeleton. Obese people with more muscular or fat tissue are wet and those with less soft tissue (called "leaner") are dry. Those who are moderate in this regard are considered as moderate in wetness/dryness (6, 9). 

Anthropometry as a branch of ergonomic science is related to the study of the weight and dimensions of the human body and in medical sciences, it is more related to the health and illness. However, anthropometric indices play a major role in Mizaj determination in PM (16-19), there are not enough studies about the relationship between the anthropometric indices and Mizaj. In this study, those anthropometric indices which were considered as a part of Mizaj determination factors in PM were extracted and their relationship with Mizaj was assessed.

## Methods

**Indices determination based on PM literatures and PM experts’ opinion:** The details of this step are fully outlined in the previously published article (3). The anthropometric indices that were mentioned in PM sources as “physique” were extracted and were examined in collaboration with a team of PM experts and they added some other indices to the list to be measured.

**Selection of participants:** 150 volunteer students of Tehran University of Medical Sciences participated in the study and their Mizajes were determined by four PM experts individually. Every PM expert determined the Mizaj of volunteers, considering two aspects of warmness/coldness (warm/cold/temperate) and wetness/dryness (wet/dry/temperate) and recorded it separately. Those participants, whose Mizajes assessment possesses **≥**70% agreement between the experts, entered the next step of the study. All steps of their selection, inclusion, and exclusion criteria are outlined in the previously published article (3). 

**Methods of measurement: **Following instruments were applied for indices measurement:

- Anthropometric board measure in dimensions of 1**×**2 meters
- Measuring tapes
- Seca digital scale with one tenth kilogram error
- In size digital caliper in large and small sizes

All volunteers were wearing stretch underwear and were in anthropometric standard position at the time of measurement. Indices were measured by a trained technician. 

**Data analysis:** In the first step, the participants in each quality of Mizaj (warmness/coldness, wetness/dryness and temperate) were put into six models of qualities and then divided into two groups. For example, in warm model, they were divided into warm and non-warm groups. The frequency distribution table of the indices was drawn and several cutoff points were considered for each index. According to each cutoff point all indices were converted to double-state variables. For example, the numbers equal to and above each cutoff point and lower of that were considered as related to warmness and coldness of Mizaj, respectively. Receiver operative characteristic (ROC) curve was drawn for variables and the cutoff points with area under curve (AUC) ≥0.6 were selected for the next step of the analysis. In the second step, the Binary Logistic Regression was used by the Forward stepwise method for each model. In the first stage, the selected cutoff points from the previous step (AUC≥0.6) were analyzed and those with acceptable p (<0.2) were kept in the model. This process was applied for other indices as well (20-22). Finally, the indices that were most associated with the defined Mizaj models were determined.

## Results

**Literature review and PM experts’ opinion:** Based on the PM literatures, the innate Mizaj which is inherently accompanied by birth is described by two qualities including hotness, coldness (hotness, coldness temperate in hotness/coldness) and wetness, dryness (wetness, dryness and temperate in wetness/dryness). Ten Mizaj determination indices were used by PM scholars to assess these two groups of qualities and then determine the Mizaj of individuals. 

Indices of body dimensions with the most consensuses in PM literatures include: chest dimensions, limb dimensions especially the palms and soles, joints and laryngeal prominence and shape of the nose (3, 9-13, 23). Big size limbs and chest, width and prominence superficial veins and prominence joints are related to warmness, small size of them is related to coldness and moderate size is related to temperate quality of coldness/warmness. Width and dilation of nostril, large size of human trunk are related to warmness and small size and shortness of fingers are related to coldness. Non-prominent joints, thickness of fingers, big eyes, broad nose, prominent cheeks, little hair of the face, thin and soft nails, high fat and muscle tissue are signs of wet Mizaj, prominent joints, larynx, narrow neck and nose, low fat and muscle tissue are seen in dry Mizaj. The mediocre of the above-mentioned indices indicate temperate Mizaj. The prominent tendons, bones and joints are a sign of warm/dry Mizaj. Obesity with predominant fat tissue indicates cold/wet Mizaj, muscular body indicates warm/wet Mizaj (6). Finally, 23 anthropometric indices were extracted. 21 indices were to be measured by the researcher, one of them (foot size) was self-reported and BMI was determined by formula: [weight (Kilogram)height Meter2. Definitions of indices based on anthropometric sources are shown in Table 1.

**Participants selecting:** Among the 150 invitees, 14 were excluded due to lack of inclusion criteria and 15 of them did not complete the study. Finally, Mizaj of 121 volunteers were determined. There was an agreement rate of ≥70% among the experts about the Mizaj of 52 volunteers (26 men with mean age of 21±1.36 and 26 women with mean age of 21±1.17). Their anthropometric indices were measured as shown in Table 1. 

**Data analysis: **After drawing the ROC curve and determining the best cutoff points as well as the repeated Binary Logistic regression, four models of warm, cold, wet and dry Mizaj were determined for each gender. Indices that had the most significant relationship with the specified Mizaj were extracted (Table 2). Based on the final extracted model, warm Mizaj in men had the most relationship with height ≥176_cm_, the chest depth at the nipples ≥197_mm_, and the palm width ≥84_mm_. In women, warm Mizaj had the most relationship with head height ≥210_mm_, shoulder width ≥369_mm_, and the sole width ≥88_mm_.In the cold model in men, the most relationship was among the Mizaj and weight ≤62_kg_, the head width ≤158_mm_, and chest depth below the nipples ≤175_mm_. In cold model in females, this significant correlation was among Mizaj and height ≤164_cm_, chest width ≤262_mm_, chest depth at the nipples ≤210_mm_, and leg width in the narrowest part ≤53_mm_.

In the wet model, wetness had the most correlation with BMI ≥22 and head circumference ≥580_mm_ in men; and with BMI ≥21, the head width ≥149_mm_, and the chest depth at the nipples ≥219_mm_ in women. 

In dry model, dryness had the most correlation with BMI ≤21, weight ≤62_kg_, and head width ≤153_mm_ in male, and also had the most correlation with BMI ≤20, the head width ≤149_mm_, and the chest depth at the nipples ≤219_mm_ in females.

**Table 1:** Definition of studied anthropometric indices and their mean in the subjects

**Female** **mean ± SD**	**Male** mean ± SD*	**Definition**	**Scale**	**Index**	
21±3	22±3	(weight (Kilogram))/[height (Meter)]^2	-	Body Mass Index	**BMI**
54±6	61±1	Body weight without shoes in fasting state	Kilogram	Weight	**A1**
160±6	175±6	Vertical distance from ground to head tip	Centimeter	Height	**A2**
549±19	593±77	Head circumference at the site of occipital bone protrusion in posterior and eyebrow in the anterior	Millimeter	Head circumference	**A3**
762±63	811±83	The size of the waist circumference at the place of the iliac crest	Millimeter	Waist circumference	**A4**
180±8	184±9	Distance between eyebrows and occipital bone in midline	Millimeter	Head anterior-posterior diameter	**A5**
151±5	157±8	Maximum head width above the ears level	Millimeter	Head width	**A6**
210±9	237±7	Distance between the chin and the top of the head	Millimeter	Head height	**A7**
373±25	426±22	Maximum horizontal width of the shoulders which is between the prominence of deltoid muscles	Millimeter	Shoulder width	**A8**
253±16	269±16	Maximum chest wall width at mid-axillary line	Millimeter	Chest width	**A9**
213±21	206±24	Maximum horizontal distance from vertical reference plain located behind the person and front of the chest at the tip of the nipple	Millimeter	Chest depth at the nipples	**A10**
155±18	188±21	Maximum horizontal distance from vertical reference plain located behind the person and front of the chest at the crease under the breast	Millimeter	Chest depth below the nipples	**A11**
75±6	83±4	Maximum width of the palm (the ends of metacarpal bones)	Millimeter	Palm width	**A12**
173±11	190±9	Distance between wrist crease and middle finger tip	Millimeter	Palm length	**A13**
50±3	59±7	Wrist width at the distal of head of the radius bone	Millimeter	Wrist width	**A14**
17±1	20±1	The width of closest joint to the palm in index finger	Millimeter	Index finger joint width	**A15**
95±6	104±5	Distance between posterior of metacarpo-phalangeal joint and index finger tip when the fingers are perpendicular to palm	Millimeter	The index finger length	**A16**
1577±110	1728±76	Maximum horizontal distance of right and left hands middle fingers’ tips when the arms are completely open	Millimeter	Distance between fingertips of both hands	**A17**
92±5	98±6	Maximum sole horizontal width perpendicular to the longitudinal axis	Millimeter	Sole width	**A18**
236±12	261±11	Parallel distance with the long axis of the sole from the back of the heel to the tip of the tallest toe	Millimeter	sole length	**A19**
64±3	71±4	The distance between the internal and external ankles	Millimeter	Ankle width	**A20**
53±4	55±5	The width of the shin at the top of the ankle in the narrowest part	Millimeter	Shin width in the narrowest part	**A21**
38±1	42±1	According to the volunteer’s report	-	Shoe size	**A22**

**Table 2:** Significant indices in warm, cold, wet and dry models of Mizaj (cutoff points)

	**Index**	**Warm Group**		**Cold Group**		**Wet Group**		**Dry Group**	
	**Gender**	**Male** ^1^	**Female** ^2^	**Male** ^3^	**Female** ^4^	**Male** ^5^	**Female** ^6^	**Male** ^7^	**Female** ^8^
**BMI**	Body Mass Index					**≥22** ^9^ P=0.04^10^ OR=13.3^11^	**≥21** P=0.02 OR=28.5	**≤21** P=0.1 OR=11.7	**≤20** P=0.04 OR=16.5
**A1**	Weight			**≤62**_kg_ P=0.07 OR=25				**≤62**_kg_ P=0.14 OR=8.8	
**A2**	Height	**≥176**_cm_ P=0.17 OR=4.8			**≤164**_cm_ P=0.11 OR=0.1				
**A3**	Head circumference					**≥580**_mm_ P=0.09 OR=6.1			
**A6**	Head width			**≤158**_mm_ P=0.07 OR=22.7			**≥149**_mm_ P=0.13 OR=9.5	**≤153**_mm_ P=0.19 OR=6.8	**<149**_mm_ P=0.03 OR=35.5
**A7**	Head height		**≥210**_mm_ P=0.1 OR=10.33						
**A8**	Shoulder width		**≥369**_mm_ P=0.08 OR=7.13						
**A9**	Chest width				**≤262**_mm_ P=0.18 OR=7.8				
**A10**	Chest depth at the nipples	**≥197**_mm_ P=0.03 OR=21.6			**≤210**_mm_ P=0.03 OR=19.9		**≥219**_mm_ P=0.07 OR=15.9		**219**_mm_ P=0.11 OR=13.1
**A11**	Chest depth below the nipples			**≤175**_mm_ P=0.05 OR=28.9					
**A12**	Palm width	**≥84**_mm_ P=0.07 OR=10							
**A18**	Sole width		**≥88**_mm_ P=0.08 OR=10.23						
**A21**	Shin width in the narrowest part				**≤53**_mm_ P=0.15 OR=7.4				

## Discussion

Standardization of the innate Mizaj indices is one of the main goals of research in PM. Accordingly, attention to indices that are less influenced by environmental factors and age change such as anthropometric indices seems essential. Although much anthropometric information is available, it seems that there is no similar study about the correlation between Mizaj and anthropometric indices. A number of studies have focused on anthropometric indices of different races in different geographic regions. For instance, Joneidi et al. (2007) evaluated the average dimensions of the anthropometric dimensions of 20-60 years old people of different ethnic groups in Iran (24). As the mean age of our participants was about 21 years, we did not find any similar study for 20-30 years old Iranians to compare it with the present study. Gharedaghi et al. (2014-2016) studied the anthropometric parameters of bone in 225 Iranian men (25). Some studies have compared the anthropometric indices in both genders. Most of the studies in medical fields concentrate on the relationship of metabolic syndromes and a few numbers of anthropometric indices (26). Some others have evaluated the relationship between anthropometric indices and lifestyles, risk factors of diseases and physiological disorders (16, 27-29). 

There are also many studies about anthropometric indices which are most related to the environmental health (30-32). Standardization of anthropometric indices in determining Mizaj as the main access in the diagnosis, treatment and prevention of diseases in PM can be an effective step in improving human health and treatment of diseases. This study seems to be remarkable for being one of the first steps in standardization of diagnostic criteria in PM. 

In the present study, in warm Mizaj model, equal or higher dimensions of the defined cutoff points of the chest depth at the nipple (OR=21.6), palm width (OR=10) and height (OR=4.8) of men had a significant correlation with warmness and head height (OR=10.3), sole width (OR=10.2) and shoulder width (OR=7.1) of women had a significant correlation as well. Based on viewpoint of PM, warm-Mizaj people have larger dimensions, especially shoulder width, chest depth, palm and sole width, and; therefore, our findings confirm this viewpoint in PM. Although height was not mention as a Mizaj index in the PM literatures, due to its commonness in related studies, its importance among the common anthropometric indices and its familiarity with people were added to the study. 

In cold Mizaj model, equal or smaller dimensions of the defined cut-off points of chest depth below the nipples (OR=28.9), weight (OR=25) and head width (OR=22) in men had a significant correlation with coldness, Chest depth at the nipples (OR=19.9), height (OR=0.1), chest width (OR=7.8) and leg width in the narrowest part (OR=7.4) in women had a significant correlation as well. These findings confirm the claim of PM literatures about the reduction of chest and limb dimensions in cold-Mizaj individuals. 

In wet Mizaj model, equal or higher dimensions of the defined cutoff points of BMI (OR=13.3) and head circumference (OR=6.1) in men had a significant relationship with wetness. BMI (OR=28.5), chest depth at the nipples (OR=15.9) and head width (OR=9.5) in women had a significant relationship as well. Meanwhile, the BMI index had the highest ratio of coefficients with the women's wet Mizaj (BMI ≥21, P=0.02, OR=28.5).

In dry Mizaj model, equal or smaller dimensions of the defined cut-off points of BMI (OR=11.7), weight (OR=8.8) and head width (OR=6.8) in men had a significant correlation with dryness. Head width (OR=35.5), BMI (OR=16.5) and chest depth at the nipples (OR=13.1) in dry-Mizaj women had a significant correlation with dryness. BMI in all wet and dry for both genders, head width in dry-Mizaj of both genders and wetness in females had high correlation with Mizaj.

Further studies need to be done to determine the more precise cutoff points of these indices to determine the warmness/coldness and wetness/dryness of the individuals. Based on PM, among anthropometric indices, it is expected that warmness/coldness of Mizaj is more indicated by bone dimensions, while wetness/dryness is more indicated by soft tissue. This viewpoint is confirmed in the framework of differences in BMI, chest, palm and sole dimensions in the current study. BMI, which is more closely related to soft tissue was associated only with wetness/dryness. Palm and sole indices which mainly represented the bone dimensions of the hands and feet were only related to the hotness/coldness. Chest and shoulder dimensions were correlated with both qualities of warmness/coldness and wetness/dryness because they are affected by both soft tissue and bone dimensions.

We had some limitations in this study including the lack of a standard tool for determining the Mizaj of volunteers by researchers and the disagreement between the PM experts in determining the volunteers’ Mizaj. Therefore, the researchers accepted those with ≥70% agreement among PM experts in their Mizaj determination as the standard group, so 69 from 121 volunteers were excluded. The third limitation was the number of volunteers with the temperate Mizaj, so they were excluded from the study. 

This study showed there was a significant relationship between some dimensions of the body and Mizaj based on the claims of PM manuscripts. Indices related to the chest, weight, head, hand and foot width had the highest relationship with warmness/coldness of Mizaj, respectively. Three indices of BMI, head width and chest dimensions had also the highest correlation with wetness/dryness of Mizaj, respectively. We recommend further studies about the relationship between Mizaj and the abovementioned indices. We hope this study and future studies provide the necessary documentation for the validation and metrification of the anthropometric indices proposed in PM for the innate Mizaj determination.
